# Editorial: Effective methods to promote appropriate use of medicines

**DOI:** 10.3389/fphar.2023.1331906

**Published:** 2023-11-21

**Authors:** Yen-Ming Huang, Yuki Kondo, Tomoya Tachi

**Affiliations:** ^1^ Graduate Institute of Clinical Pharmacy, College of Medicine, National Taiwan University, Taipei City, Taiwan; ^2^ School of Pharmacy, College of Medicine, National Taiwan University, Taipei City, Taiwan; ^3^ Department of Pharmacy, National Taiwan University Hospital, Taipei City, Taiwan; ^4^ Department of Clinical Chemistry and Informatics, Graduate School of Pharmaceutical Sciences, Kumamoto University, Kumamoto, Kumamoto, Japan; ^5^ Department of Clinical Pharmacy, Graduate School of Pharmaceutical Sciences, Nagoya City University, Aichi, Japan

**Keywords:** adverse drug reaction, medication adherence, monitoring, pharmacist, technology

The importance of using medications appropriately cannot be overstated when it comes to effectively managing diseases and achieving positive health outcomes (Kim et al., 2018). Unfortunately, over the past two decades, there has been a global problem with the improper use of medications, particularly among older adults (Rochon et al., 2021). Research has shown that a substantial portion of older adults, ranging from 24% to 72%, are using potentially inappropriate medications (PIMs) (Tachi et al., 2019; Malakouti et al., 2021). This misuse is largely driven by two main factors: polypharmacy and the prescription of PIMs in individuals with multiple health conditions (Curtin et al., 2019). Improper medication use among older adults has serious consequences, including an increased risk of adverse drug events, unplanned hospitalizations, rising healthcare costs, and frequent visits to the emergency department (Curtin et al., 2019; Katsuno et al., 2021; Matsuyama et al., 2021). Additionally, self-medication and medication non-adherence further compromise patient health outcomes. Despite efforts to develop tailored interventions, the improvement in appropriate medication use has been slower than anticipated. One significant reason for this slow progress appears to be the absence of a thorough grasp of medication misuse and improper use, which is essential for crafting effective interventions (Costa et al., 2015).

It is worth highlighting that the benefits of using medication appropriately rely on accurate diagnosis, precise prescription, patient adherence to medication instructions, and regular monitoring by healthcare professionals. This Research Topic addresses the strategies to encourage proper medication use, considering a wide array of perspectives across various countries.

The initial step in understanding the challenges related to comprehending and effectively using medication information for making informed health decisions is to assess the user perception of this information. Alongside medication counseling, prescription drug label (PDL) instructions play a crucial role in guiding patients to adhere to their prescribed medications (Shiyanbola et al., 2016). When patients struggle to comprehend label instructions, it can lead to medication errors, reduced treatment effectiveness, and medication non-adherence. Schackmann et al. conducted a study to assess the comprehensibility of PDL instructions, comparing those presented on a personalized medication overview to the standard instructions found on medication packaging. The results showed that those exposed to the overview had a higher percentage of correct answers, particularly regarding medications with complex instructions. This study suggests that a medication overview with additional information can enhance patients’ understanding and adherence to medication instructions (Schackmann et al.). Another study examined the preferences of caregivers of children regarding accessible information on safe medication use for children, considering content, channels, and formats of healthcare information (Xu et al.). The findings indicated that caregivers primarily obtain information from medical institutions, healthcare professionals, and personal media. They favor text, pictures, and videos as content formats and prioritize the popularization of knowledge about safe medication for children (Xu et al.). Both studies address that to promote safe medication use, it is essential to disseminate accurate and comprehensible healthcare information about medications through diverse channels, accounting for user preferences.

Ensuring proper medication use also hinges on accurate prescribing to prevent serious adverse drug reactions (ADRs). Liu et al. conducted a retrospective analysis of inpatient prescribing practices for long-acting injectable (LAI) antipsychotics and their oral or short-acting injectable (SAI) equivalents in patients with psychological disorders. They discovered that LAIs were generally underutilized when compared to their oral or SAI counterparts from 2010 to 2016. The variations in antipsychotic prescribing patterns provide valuable insights for future research aimed at understanding the reasons behind disparities in medication utilization (Liu et al.). In another study, Shang et al. and their team performed a network meta-analysis involving 159 randomized controlled trials to evaluate the efficacy of pharmacological interventions for smoking cessation. Their findings suggested that varenicline was more effective than other monotherapies, and combination interventions outperformed monotherapy. Whether used as monotherapy or in combination, these interventions showed benefits for smoking cessation compared to a placebo (Shang et al.). Additionally, a separate network meta-analysis of 33 randomized controlled trials compared various pharmacological interventions for preventing opioid-induced hyperalgesia and its impact on postoperative pain. This analysis found that amantadine was the most effective in reducing postoperative pain intensity, while dexmedetomidine produced the best results in reducing the incidence of postoperative nausea and vomiting (Xie et al.). These findings, derived from longitudinal data and meta-analysis, provide valuable guidance for selecting medications to achieve better pharmacological effects while minimizing associated adverse effects.

Implementing drug monitoring as part of medication management aids in the identification of factors that may lead to ADRs and enables early intervention. A review of 26 articles investigated sex differences in ADRs associated with commonly used psychotropic, cardiovascular, and analgesic medications (Shan et al.). The findings pointed out over half of these studies revealed sex-specific patterns in ADR occurrence. Some severe ADRs exhibited variations related to sex, such as a higher prevalence of clozapine-induced neutropenia in women and a more pronounced incidence of liver function issues with simvastatin/atorvastatin in men. Consequently, accounting for sex differences in ADRs may be a critical consideration for clinical decision-making (Shan et al.). Cai et al. examined the current status of individualized pharmaceutical care in China, which includes therapeutic drug monitoring (TDM), pharmacogenetic (PGx) testing, and pharmacist-managed clinics. Their findings indicated that only a small percentage of hospitals conducted TDM and performed PGx testing. This study underlines the early stage of development and emphasizes the necessity for collaboration across various sectors to establish comprehensive individualized pharmaceutical care (Cai et al.). In the context of monitoring ADRs and addressing drug-related concerns, one study introduced a pharmacist-led olaparib follow-up program for patients with ovarian cancer, offering patient education and proactive monitoring (Wang et al.). The findings revealed common ADRs occurring early in treatment, allowing for medication adjustments. Additionally, pharmacists identified clinically significant drug-drug interactions, particularly in patients using multiple medications concurrently. This program effectively managed ADRs, optimized medication use, and improved patient care, providing valuable insights for follow-up services for patient care. In summary, increased awareness of risk factors associated with medication use and the implementation of drug monitoring and follow-up procedures enhance the early detection and mitigation of medication-related adverse events.

Medication non-adherence has consistently been identified as a fundamental challenge contributing to improper medication use. In a cross-sectional study conducted in China involving older adult stroke survivors, it was revealed that more than half of the participants displayed medication non-adherence (Cao et al.). This non-adherence was associated with lower educational levels, a higher number of prescribed medications per day, and specific concerns regarding medications. Conversely, higher health literacy and positive beliefs about medication were linked to improved adherence (Cao et al.). Another study in China focused on assessing adherence to infliximab treatment among patients with Crohn’s disease and its correlation with medication beliefs (Li et al.). It found that lower concerns about medication beliefs were associated with better adherence. Factors such as gender, marital status, travel time to the infusion center, and accommodation accessibility also influenced adherence (Li et al.). These findings draw attention to developing interventions that target the factors contributing to medication non-adherence within high-risk populations. While medication non-adherence significantly impacts the effectiveness of chronic therapy and the sustainability of healthcare systems, Medication Adherence-Enhancing Interventions (MAEIs) are underutilized and rarely reimbursed. A study examined reimbursed MAEIs across 12 European countries and found that these interventions commonly focused on adherence through approaches such as education, medication regimen management, and adherence monitoring feedback, with limited utilization of technology-mediated interventions. Wider adoption and reimbursement of MAEIs are crucial to effectively combat medication non-adherence (Kardas et al.).

Pharmacists stand out as highly accessible and well-qualified healthcare practitioners, offering a spectrum of preventive measures, ranging from primary to tertiary, for managing diseases across various healthcare settings (Shiyanbola and Huang, 2020). An integral part of enhancing medication appropriateness is the strategic integration of pharmacists into patients’ medication management, fostering collaboration with other healthcare professionals (Huang et al., 2022). Within the realm of community pharmacy, Algarni et al. adopted a qualitative approach to delve into the viewpoints of Saudi community pharmacists regarding the misuse and abuse of over-the-counter (OTC) medications. Their investigation pinpointed commonly misused OTC products, often stemming from unprofessional guidance, a lack of patient awareness, and the influence of OTC advertising. Pharmacist competence comes to the fore in addressing misuse and abuse complexities by understanding customer behaviors and employing counseling skills to recommend appropriate OTCs and alleviate ailments. Alongside a comprehensive review of OTC regulations, Algarni et al. advocate for enhanced pharmacist training and patient education to curb OTC misuse and abuse (Algarni et al.). In the context of hospital practice, a study conducted in Pakistan shed light on the perceptions of healthcare professionals and patients concerning the involvement of pharmacists in tuberculosis management (Atif et al.). Physicians acknowledged pharmacists as highly qualified healthcare professionals, while patients mainly viewed them as dispensers. Both groups reached a consensus on the potential roles pharmacists could play, such as monitoring, counseling, medication selection, dosage adjustment, and polypharmacy assessment. Physicians, facing heavy workloads, expressed readiness to delegate specific duties to pharmacists, provided they received appropriate training. However, the broader adoption of expanded pharmacist roles faces challenges such as limited interest from regulatory authorities and policymakers and concerns about pharmacist competence (Atif et al.). Meanwhile, a study in Lebanon collaborated with a multidisciplinary team of healthcare professionals to develop a pharmacist-led medication review service with a focus on deprescribing in a care facility catering to low-income patients who received free medications (Alaa Eddine et al.). This intervention effectively identified problems related to medications and generated recommendations for physicians, with a notable 30% of these recommendations being accepted. Patients who received this intervention reported significantly higher satisfaction levels compared to those who received routine care (Alaa Eddine et al.). These studies collectively underscore the competence of pharmacists in delivering pharmaceutical care and collaborating with other healthcare team members to ensure the safe and effective use of medications.

The judicious application of digital technology can play a crucial role in ensuring the appropriate use of medications, especially in the context of a pandemic. An innovative study introduced a model for an internet hospital pharmacy service in China, driven by artificial intelligence (AI), with the aim of augmenting pharmacy services amid the COVID-19 pandemic (Bu et al.). This model seamlessly integrated AI for reviewing prescriptions, an offline self-pick-up system that utilized QR codes, and AI-powered medication consultations. The AI system exhibited a commendable success rate in reviewing prescriptions, and patients expressed a preference for the offline self-pick-up system. Notably, the study revealed the popularity of medication consultations outside regular working hours, with topics ranging from the dispensing process to disease diagnosis and patient education (Bu et al.). The utilization of technology throughout the patient care journey holds the potential to ensure the safe use of medications, enhance the efficiency of pharmacy workflow, and elevate the quality of pharmacy services, particularly in the midst of a pandemic.

In summary, promoting appropriate medication use is vital for effective disease management. This Research Topic has highlighted common challenges in the course of medication use, including medication misuse, labeling, prescribing, monitoring, and non-adherence. Pharmacists play a crucial role in proper medication use, while digital technology, exemplified by AI-driven pharmacy services, enhances healthcare during crises. These multifaceted strategies aim to ensure safe and effective medication use, making strides toward improving patient outcomes and reducing the global problem of medication misuse.
